# Analysis of Precore/Core Covariances Associated with Viral Kinetics and Genotypes in Hepatitis B e Antigen-Positive Chronic Hepatitis B Patients

**DOI:** 10.1371/journal.pone.0032553

**Published:** 2012-02-27

**Authors:** Chun-Pei Cheng, Pei-Fen Lee, Wen-Chun Liu, I-Chin Wu, Chu-Yu Chin, Ting-Tsung Chang, Vincent S. Tseng

**Affiliations:** 1 Department of Computer Science and Information Engineering, National Cheng Kung University, Tainan, Taiwan; 2 Institute of Medical Informatics, National Cheng Kung University, Tainan, Taiwan; 3 Department of Biotechnology, Ming Dao University, Changhua, Taiwan; 4 Department of Internal Medicine, National Cheng Kung University Hospital, Tainan, Taiwan; 5 Graduate Institute of Clinical Medicine, National Cheng Kung University, Tainan, Taiwan; 6 Institute of Basic Medical Sciences, National Cheng Kung University, Tainan, Taiwan; 7 Infectious Disease and Signaling Research Center, National Cheng Kung University, Tainan, Taiwan; Institut Pasteur, France

## Abstract

Hepatitis B virus (HBV) is one of the most common DNA viruses that can cause aggressive hepatitis, cirrhosis and hepatocellular carcinoma. Although many people are persistently infected with HBV, the kinetics in serum levels of viral loads and the host immune responses vary from person to person. HBV precore/core open reading frame (ORF) encoding proteins, hepatitis B e antigen (HBeAg) and core antigen (HBcAg), are two indicators of active viral replication. The aim of this study was to discover a variety of amino acid covariances in responses to viral kinetics, seroconversion and genotypes during the course of HBV infection. A one year follow-up study was conducted with a total number of 1,694 clones from 23 HBeAg-positive chronic hepatitis B patients. Serum alanine aminotransferase, HBV DNA and HBeAg levels were measured monthly as criteria for clustering patients into several different subgroups. Monthly derived multiple precore/core ORFs were directly sequenced and translated into amino acid sequences. For each subgroup, time-dependent covariances were identified from their time-varying sequences over the entire follow-up period. The fluctuating, wavering, HBeAg-nonseroconversion and genotype C subgroups showed greater degrees of covariances than the stationary, declining, HBeAg-seroconversion and genotype B. Referring to literature, mutation hotspots within our identified covariances were associated with the infection process. Remarkably, hotspots were predominant in genotype C. Moreover, covariances were also identified at early stage (spanning from baseline to a peak of serum HBV DNA) in order to determine the intersections with aforementioned time-dependent covariances. Preserved covariances, namely representative covariances, of each subgroup are visually presented using a tree-based structure. Our results suggested that identified covariances were strongly associated with viral kinetics, seroconversion and genotypes. Moreover, representative covariances may benefit clinicians to prescribe a suitable treatment for patients even if they have no obvious symptoms at the early stage of HBV infection.

## Introduction

Chronic hepatitis B virus (HBV) infection has been considered to be a high mortality disease worldwide. To date, over three hundred million people have died from primary adverse outcomes including cirrhosis and hepatocellular carcinoma (HCC), which is one of the most common primary liver malignancy [1]–[3]. A large number of observations showed that high levels of viral loads in serum would profitably promote the progression of chronic hepatitis B infection. Elevated serum HBV DNA can be regarded as an indicator of human cirrhosis [4], and independently associated with HCC [5]. Three widely accepted phases of chronic hepatitis B infection have been identified based upon serum alanine aminotransferase (ALT) activity, the presence of hepatitis B e antigen (HBeAg) and HBV DNA level: (i) immune tolerant phase (ii) immune active (clearance) phase, and (iii) inactive phase [6], [7]. Although most people are persistently infected with HBV, the kinetics in serum levels of viral loads and the host immune responses vary from person to person. For instance, according to the levels of serum viral loads, patients with stationary pattern maintained stable HBV DNA levels with fluctuations of less than 1.5 log copies/ml, whereas the remaining subjects belong to the fluctuating pattern, which can be further separated into declining and wavering pattern [8]. Previous computational studies on hepatitis-related viruses have led to the development of mathematical approach to modeling the viral kinetics [9]–[11]. In our previous study, we have also developed a regression model to predict the probability of HBeAg-seroconversion in patients with HBeAg-positive chronic hepatitis B [8]. However, the viral kinetics in response to the host immune responses has yet been elucidated completely.

Four overlapping open reading frames (ORFs) within the HBV genomic sequence that can be translated to viral core proteins or HBc particles, surface proteins, reverse transcriptase and HBx [12]. Some studies have proven that the HBeAg and hepatitis B core antigen (HBcAg) encoded by HBV precore/core ORF are used clinically as indicators of HBV replication [13], [14]. Precore/core mutations of HBV genome may cause amino acid changes of the resultant protein HBeAg or HBcAg. For example, two classical mutations including G1896A and G1898A within precore/core gene region would change amino acid W28Stop and G29S respectively [15] and they are also highly related to the hepatomas [16]. Specific mutations in the precore/core region of HBV enable virus to escape the host immune system's recognition, such as cell-mediated and humoral immune attack [17]–[20]. These mutations are subsequently related to the severity of liver disease [1], [12], [21]. Besides, variant HBV genotypes (A to H, according to geographic distribution) can also cause various degrees of liver damage and viral persistence. It has been reported that patients infected with HBV genotype B are associated with earlier HBeAg-seroconversion [22], whereas the genotype C has a higher risk of cirrhosis and HCC development compared to the genotype B [23].

A potentially powerful analysis to differentiating clinical outcomes of HBV infection at an early stage is dominant, allowing patients to receive an effective treatment and a suitable antiviral therapy. The clinical outcomes of viral infection are the effect of the interaction of viruses and their hosts [20]. Referring to literature, Hosono et al. had indicated 13 positions with high mutation frequencies called mutation hotspots among 21 different HBV precore/core amino acid sequences in hepatomas [16]. The identified mutation hotspots have a pleiotropic effect on both immune and humoral immune responses, and their results also suggested that the mutation codon 159 appears to correlate very well with the mutation at codon 126. Recently, a similar result from the genome-wide amino acid analysis of hepatitis C virus showing that the covariances, derived from the high probability of interactions of amino acid residue pairs, are very different in response and nonresponse to interferon-based therapy [24]. In the past decade, the covariance analysis has been used to identify the residue pairs directly interacting in protein 3-dimentional structures [25], to examine the functionally important residues in proteins [26], and to infer protein-protein interactions [27]. However, the analysis of covariance in viral proteins in response to patient serological or virological course of infection has yet been conducted. It seems as if the existence of certain diverse genetic interactions may be regarded as clinical indicators during the course of viral infection.

In this study, we investigated if certain variations of HBV precore/core encoding 212 amino acids (the regions of precore and core have 29 and 183, respectively) were associated with the viral kinetics, seroconversion or genotypes in treatment-naïve chronic B patients during a one-year follow-up. Notably, covariance analysis does not take into consideration on temporal component of our sequence data, and therefore it is not a suitable method to be applied directly. The results of mining single-point mutations [28] varied with time of each residue were identified and then utilized to make the developed method available for the time-varying sequences, leading to an increase in the precision of hotspots identification; hence, the resultant time-dependent covariances may permit indicators of serological and virological outcomes of HBV-infected patients.

## Methods

### Ethics statement

This study was verified and qualified by the Institutional Review Board of National Cheng Kung University Hospital from 1/17/2011 to 7/31/2012 under contract number “ER-99-386”. The ethics committee specifically waived the need for informed consent forms since the data were publicly obtained from an observational study and analyzed anonymously.

### Patients and study design

A one year follow-up study enrolling treatment-naive 23 patients with HBeAg-positive chronic B in genotype B (8 patients) and C (15 patients) from National Cheng Kung University Hospital was conducted. A serum ALT level of less than 10 times of the upper limit of normal (ULN), HBV DNA level at least than 1 million copies/ml, bilirubin level of less than 2.5 times of ULN, the detectable of hepatitis B surface antigen (HBsAg) for at least the previous 6 months, and the detectable of HBeAg were examined at the time of screening. If the patients were infected by hepatitis C or D viruses, had inactive HBsAg, immunodeficiency virus infection, autoimmune hepatitis, decompensated liver disease, or treated with systemic antiviral therapy, immunomodulators, cytotoxic agents or corticosteroids, they would be excluded in this study.

All the patients received placebos and had regular follow-ups for 1 year. Serum ALT, bilirubin, and HBV DNA levels were monthly checked for all enrolled patients. According to the time-varying serum viral load, eligible patients were divided into two major subgroups including stationary pattern (6 patients) and fluctuating pattern (17 patients). Patients with stationary pattern have a stable HBV DNA level with fluctuations of less than 1.5 log copies/ml during this one year period. The patients with fluctuating pattern could be further divided into declining pattern (9 patients) and wavering (8 patients) pattern. The more detailed subgrouping criteria could refer to our previous work [8]. The variables of clinical course and outcome among patients categorized by patterns of viral kinetics were shown in Table 1. Alternatively, as with the same subgroup criteria presented in our previous work [8], seventeen patients with fluctuating pattern also could be categorized into two subgroups including HBeAg-seroconversion (6 patients) and HBeAg-nonseroconversion (11 patients) based on the detectable antibody of HBeAg in serum leading to a year-end decrease in the level of HBeAg. Although the remaining 6 patients with stationary pattern also have undetectable antibodies, they were still not categorized into the subgroup of HBeAg-nonseroconversion since they have continuously stable serum viral loads and ALT during the follow-up. The variables of clinical course and outcome among these patients were shown in Table 2.

**Table 1:** Variables of clinical course and outcome among patients categorized by patterns of viral kinetics.

Variables	Stationary pattern	Fluctuating pattern	Declining pattern	Wavering pattern
Number of patients	6	17	9	8
Sex (male∶female)	2∶4	13∶4	6∶3	7∶1
Age (years)	36.17±3.66	34.12±9.51	34.11±11.15	34.13±8.03
Baseline bilirubin (×ULN)	0.6±0.24	0.71±0.26	0.64±0.17	0.78±0.33
Baseline ALT (×ULN)	1.32±0.45	4.13±2.78***	4.28±2.45**	3.97±3.27
Baseline HBV DNA (log copies/ml)	8.82±0.21	7.75±1.23**	7.92±1.02*	7.56±1.47*
Baseline necroinflammatory score	2.83±1.33	7.76±2.8***	7.67±3.35**	7.88±2.23***
Baseline fibrosis score	0.17±0.41	2.06±1.2***	1.89±1.36**	2.25±1.04***
Final bilirubin (×ULN)	0.58±0.20	0.67±0.26	0.56±0.21	0.78±0.27
Final ALT (×ULN)	1.91±1.57	1.44±1.48	0.95±0.60	1.99±1.99
Final HBV DNA (log copies/ml)	8.91±0.47	5.59±1.29***	4.91±1.14***	6.34±1.05***
Final necroinflammatory score	4.67±1.97	5.88±2.90	5.11±2.62	6.86±3.13
Final fibrosis score	0.67±0.52	2.06±1.44**	1.78±1.48	2.43±1.40*
Genotypes (B∶C)	3∶3	5∶12	1∶8	4∶4

Abbreviations: HBeAg: Hepatitis B e antigen; ALT: Alanine aminotransferase; ULN: Upper limit of normal; HBV: Hepatitis B virus.

*: P<0.05 compared with the stationary pattern.

**: P<0.01 compared with the stationary pattern.

***: P<0.001 compared with the stationary pattern.

**Table 2:** Variables of clinical course and outcome among patients categorized by HBeAg-seroconversion.

Variables	Patients with seroconversion	Patients without seroconversion	P value
Number of patients	6	11	-
Sex (male∶female)	5∶1	8∶3	0.622
Age (years)	30.67±12.77	36±7.21	0.377
Baseline Bilirubin (×ULN)	0.76±0.1	0.68±0.32	0.42
Baseline ALT (×ULN)	3.04±2.19	4.73±2.97	0.205
Baseline HBV DNA (log copies/ml)	7.58±1.57	7.84±1.07	0.722
Baseline Necroinflammatory score	6.33±3.01	8.55±2.46	0.159
Baseline Fibrosis score	1.5±1.22	2.36±1.12	0.184
Final bilirubin (×ULN)	0.68±0.15	0.66±0.30	0.942
Final ALT (×ULN)	0.85±0.33	1.78±1.76	0.105
Final HBV DNA (log copies/ml)	4.84±1.29	6.15±1.09	0.009
Final necroinflammatory score	5.33±3.39	6.1±2.56	0.731
Final fibrosis score	2.33±1.51	1.90±1.45	0.584
Genotypes (B∶C)	3∶3	2∶9	0.169

Abbreviations: HBeAg: Hepatitis B e antigen; ALT: Alanine aminotransferase; ULN: Upper limit of normal; HBV: Hepatitis B virus.

During the one-year follow-up period, multiple full-length HBV precore/core ORFs were sequenced monthly via clone sequencing from the all 23 qualified patients. These DNA sequences were directly in-frame translated into sequences of amino acid using ExPASy translation tool (http://au.expasy.org/tools/dna.html). Eventually, a total number of 1,694 amino acid sequences were conducted as the input data of this study. As stated in the previous two paragraphs, for each subgroup, time-dependent covariance analysis were conducted on their corresponding time-varying sequences. Several distinct covariances were presented by four different comparisons including the stationary versus fluctuating patterns, declining versus wavering patterns, HBeAg-seroconversion versus HBeAg-nonseroconversion, and genotype B versus genotype C. The detailed procedure for the analysis would be introduced in a later paragraph.

### PCR amplification of HBV precore/core ORF and genotyping

The serum HBV DNA was extracted with a Viogene extraction kit, and real-time PCR was then performed on a LightCycler instrument (Roche Diagnostics Applied Science) using primers and probes. The genotyping method was based on melting curve analysis (MCA) with LightCycler hybridization probes [29]. The PCR reaction was run in a total volume of 10 µl, containing 2.5 µl of DNA template, 1 µl of LightCycler FastStart DNA Master Hybridization Mixture (Taq DNA polymerase, PCR reaction buffer, 10 mM MgCl2, and dNTP mixture) (Roche Diagnostics Applied Science), 1.2 µl of 25 mM MgCl2, 0.075 µl of 20 µM each of the probes, and 0.5 µl of 5 µM of each primer. The amplification using set 1 (ACDG/BEF set) and set 2-1 (B/E/F set) amplicons was performed as follows: initial hot start denaturation at 95°C for 10 min, which was followed by 45 cycles of denaturation at 95°C for 10 s, annealing at 53°C for 10 s, and extension at 72°C for 15 s. The programmed temperature transition rate was 20°C/s for denaturation/annealing and 3°C/s for extension. Real-time PCR monitoring was achieved by measuring the fluorescence at the end of the annealing phase for each cycle. After PCR, a melting curve was generated by holding the reaction at 95°C for 10 s and then lowering the temperature to 48°C at a transition rate of 20°C/s and holding for 60 s. It was then followed by heating slowly at a transition rate of 0.1°C/s to 80°C with continuous collection of fluorescence at 640 nm. The melting curve and quantitative analysis were conducted by using LightCycler analysis software version 3.5 following the manufacturer's instructions (Roche Diagnostics Applied Science). For set 2-2 amplicon (A/CD/G set+C/D set), the PCR reaction was the same as that of set 1 and set 2-1, except for the extension at 72°C for 20 s.

### Cloning process

HBV DNA was amplified by PCR with commercially available Herculase enhanced DNA polymerase (*Stratagene*). Primers amplify the precore/core ORF of the HBV strains. The cycling conditions of PCR were 3 minutes at 94°C followed by 35 cycles of 40 seconds at 94°C, 50 seconds at 60°C and 1 minute at 72°C. The PCR products were separated by 1% agarose gel electrophoresis and purified for sequence analysis using the QIAGEN QIA Quick Gel Extraction Kit. Purified PCR products were cloned into a pGEM plasmid and then analyzed by sequencing of 20 clones using the Applied Biosystems ABI PRISM Big Dye Terminator Cycle Sequencing Ready Reaction Kit. An automated DNA sequencer ABI 310 was used to determine amplified DNA sequences.

### Time-dependent covariances identification

Time-dependent covariances can be generated by two computational steps. For each subgroup, covariance analysis is firstly applied on the patients' corresponding time-varying HBV precore/core amino acid sequences. Then the identified covariances will be filtered by mining single-point mutations of each residue that do not vary with time. The remaining set containing covariances from the filtering process was called “time-dependent covariances”.

The following equations below show you how to identify the covarying pairs of residues.
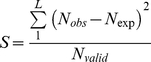
(1)In Eq. (1), *S* represents the degree of covariance between two columns (positions) in a set of aligned amino acid sequences. *L* is the list number of all observed pairs and *N_obs_* is the number of occurrences for the pair of residues. The expected number, *N_exp_*, for the pair as shown in Eq. (2).
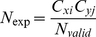
(2)


In Eq. (2), *N_valid_* is the number of sequences within a sequence set, *C_xi_* is the observed number of residues *x* at position *i*, and *C_yj_* is the observed number of residues *y* at position *j*. For example, given that a patient subgroup has only 4 (*N_valid_* = 4) aligned sequences having 5 amino acids at two different time points, t_1_ = {LNVIV and IYVLG} and t_2_ = {IYVLG and VYVLG}. The covariance of position 1 (*x* = 1) and 5 (*y* = 5) can be calculated by the following steps: Since covariance analysis is only available on handling a general set of sequences, i.e., without considering the order of sequences being analyzed, the input sequences are subsequently put together as a new set, {LNVIV, IYVLG, IYVLG and VYVLG}. Position 1 has three distinct amino acids, L (*C_1L_* = 1), I (*C_1I_* = 2) and V (*C_1V_* = 1), and position 5 has two, V (*C_5V_* = 1) and G (*C_5G_* = 3). From combinational pairing of these two positions, six (*L* = 6) observation pairs including LV, LG, IV, IG, VV and VG can be generated. Mapping onto the original 4 sequences, IG appears twice, LV and VG appear once, and LG, IV and VV do not appear at all. Thus, score *S* of these two positions is about 0.44. The detailed computational process is given by the following formula.
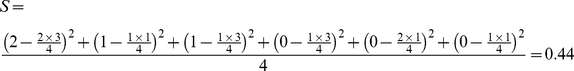



After iteratively calculating covariance scores of all possible residue pairs, a user-set threshold can be used to judge whether there truly exists covariance of a residue pair. In this study, the threshold was set as 0.5. For each pair, if its score below 0.5, there does not exist a covariance. However, how to set an appropriate threshold is a challenge. Two statistical methods were used to examine it as shown in the part of Statistical analysis.

The identified covariances were further filtered by the results of single-point mutations to derive time-dependent covariances. An example of mining single-point mutations was shown in Figure S1. Given that a patient subgroup has 5 equal-length sequences at each time point, spanning from t_1_ to t_4_. Conserved sequences, CS_1_ to CS_4_, of each time point will be subsequently identified. For each position, residues with different amino acids at two consecutive time points will be mined. From looking at the mining results listed at the bottom of the figure, “4: t_1_ (D)→t_2_ (H)” represnts that a residue with D turns into H at position 4 from t_1_ to t_2_. The same concept can be applied on the remaining rules. Finally, if residues covary in the identified covariances via Eq. (1) and (2) as well as do not appear in the list of point-mutation rules (these residues do not change over time), it will be eliminated from the first step-derived covariances. In other words, the preserved residues not only have covarying characteristics but also alter the properties of amino aicds over time. These filtered covariances were called “time-dependent covariances”.

### Leave-one-out-like cross-validation

**Figure 1:**
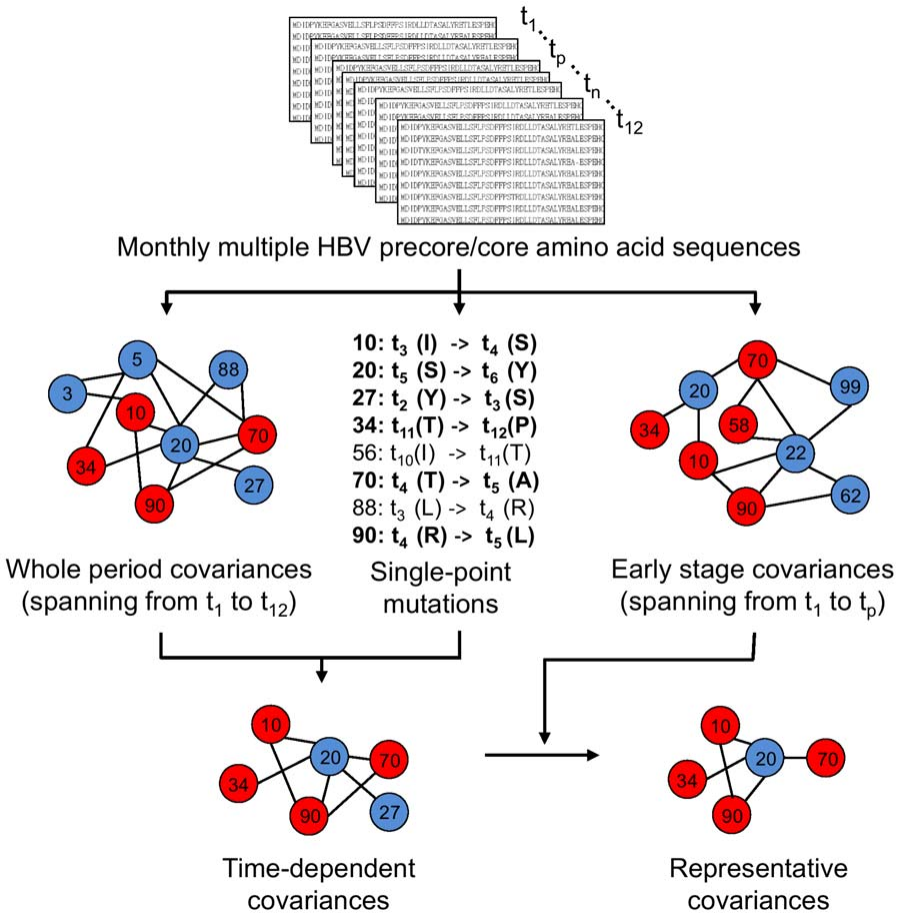
Schematic overview of data step processing. Data processing flowchart is composed of four steps from top to bottom. First, the input data is one subgroup corresponding set of HBV time-varying amino acid sequences. Second, whole period covariances, early stage covariances and single-point mutations varied with time are identified as shown in the middle part of the figure. Third, through filtering whole period covariances with single-point mutation rules, time-dependent covariances can be derived. Finally, representative covariances of each subgroup can be generated via determining the intersection of time-dependent and early stage covariances. t_1_, t_p_, t_n_ and t_12_ stand for time points at baseline, peak, nadir and year-end viral loads respectively.

Representative covariances of each subgroup were examined by using a proposed leave-one-out-like cross-validation method. An example showed in Figure S2 illustrates how to perform the evaluation process. Given that a patient subgroup involves 3 patients. The sequences derived from each one of the patients will be regarded as the testing data, and the remaining subjects are belonging to the training data. The covariances can be identified through a complete analysis flow showed in Figure 1. If covariances of testing data are covered by that of its corresponding training data, the covariances will be preserved as shown at the bottom of the figure. In this example, there are 2 patients can be covered by covariance 116–126 and 126–159; thus its coverage rate is about 67% (2/3).

### Statistical analysis

In Table 1 and Table 2, the frequencies and distributions of categorical variables for all patients were compared by Chi-square test. Continuous variables were compared by Student's t-test.

For determining an appropriate threshold of covariance analysis, we set same, 0.5, as a previous work by Aurora et al. [24]. In Table S1, two statistical examinations including McNemar's test and Permutation test were performed to test the significance of identified covariances of each patient subgroup. Thirteen mutation hotspots of HBV precore/core amino acids in hepatomas proven by Hosono et al. [16] were curated as a ground truth. These hotspots were listed in Text S1. McNemar's test was utilized to confirm whether distributions and frequencies of categorical variables (hotspots) are associated with covariances. Then permutation test was used to examine whether ratios of hotspots to covarying residues are larger than a random condition. In brief, we defined R_hotspots/residues|covariances_ as a ratio of the number of hotspots to all covarying residues, and R_hotspots/residues|random_ as a random distribution ratio of the number of hotspots to a full-length, 212 residues, HBV precore/core amino acid sequence. A null hypothesis R_hotspots/residues|random_> = R_hotspots/residues|covariances_ by performing 1,000 resampling was tested. Permutation test P value is the proportion of these resamples that give a result the null hypothesis is true. The results are considered statistically significant as long as P<0.05 from any one of two tests.

## Results

### An overview of data step processing

Monthly derived multiple HBV precore/core amino acid sequences of each patient were prepared as input data for mining covariance and single-point mutation rule. A schematic overview of the study was shown in Figure 1. Covariances of each subgroup could be derived by applying covariance analysis directly on their corresponding sequences such as a whole period (middle left in Figure 1) and an early stage (middle right in Figure 1). The covariances were visually presented in a network, in which the nodes represented covarying amino acid residues. If there is a covariance of two residues, they will be linked by an edge. Literature-reported mutation hotspots were shown in red, whereas the remaining nodes were shown in blue. Then the single-point mutations would be mined from the time-varying sequences. A simple list of point mutation rules has been shown in the middle of the figure. Each rule consisted of position number of residues and one-letter codes of amino acids at two time points. Twenty kinds of amino acids were divided into 4 subgroups based on their side-chain properties including non-polarity (G, A, V, L, I, F, W, M and P), neutrality (Y, N, Q, S, T and C), acidity (D and E) and alkalinity (H, K and R). The rules would be marked in bold if their changes involve two different properties. Only bold mutation rules will subsequently be used to screen the aforementioned whole period covariances, namely time-dependent covariances. Diverse time-dependent covariances of each subgroup may provide insights into residue interactions in responses to serum viral kinetics, seroconversion and genotypes during the course of HBV infection.

The most important issue is how clinicians prescribe patients a good diagnosis or a suitable treatment at an early stage of HBV infection even if they exhibit no obvious symptoms. Hence, for each subgroup, covariances at an early stage spanning from baseline, t_1_, to a peak of viral load, t_p_, were used to determine the intersections with their corresponding time-dependent covariances. These preserved covariances are shown in the bottom right of Figure 1. In this study, a proposed leave-one-out-like cross-validation was used to develop the representative covariances of each subgroup that visually present in a tree-based structure. It may help clinicians to predict the serological or clinical outcomes at an early stage of HBV infection.

### An improvement to increase the proportion of mutation hotspots

**Figure 2:**
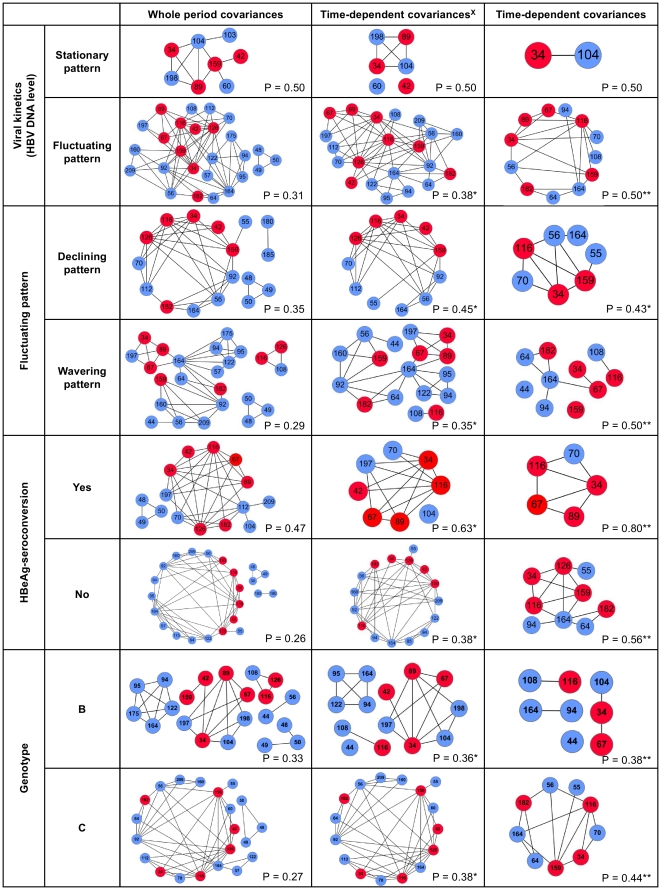
Comparisons of covariances in all subgroups. Left column shows the covariances derived from a set of whole period sequences. The time-dependent covariances are shown in the middle and right columns. ^X^: Residues changed with time regardless of amino acid properties. P: Proportion of mutation hotspots of covariances. *: Proportion of mutation hotspots greater than left column. **: Proportion of mutation hotspots greater than middle column.

To check whether covariances derived from HBV precore/core amino acid sequences are associated with serum viral kinetics, seroconversion and genotypes, Figure 2 presents three kinds of covariances of each subgroup, including whole period covariances (left column), time-dependent covariances (right column) and time-dependent covariances regardless of amino acid properties (middle column). All of the single-point mutations to time-dependent covariances were shown in Table S2 and the detailed rules in Table S3. Moreover, consulting previously identified HBV infection-related mutation hotspots in hepatomas [16], 13 residues (Text S1) were curated to observe hotspot distributions in our results. Intriguingly, most subgroups showed higher proportions of mutation hotspots filled in red that was remarkable in the time-dependent covariances compared to the other two (left and middle column).

**Figure 3:**
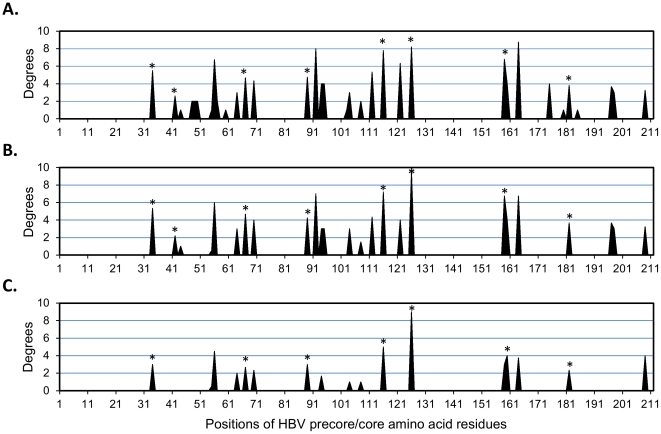
Average degrees of each position of three kinds of covariances identification method. X-axis represents amino acid positions of a full length of HBV precore/core amino acid sequences. Y-axis represents average numbers of degrees. A) Average degrees of positions of whole period covariances. B) Average degrees of positions of time-dependent covariances regardless amino acid properties. C) Average degrees of positions of time-dependent covariances. *: Literature-reported mutation hotspots.

In addition, the average number of degrees of every position (node) was calculated from 8 covariance networks involved in the same column in order to achieve an objective comparison of the covariances displayed in Figure 2. The average degree of each amino acid position showed a similar trend in Figure 3. For instance, in Figure 3C, seven out of seventeen covarying residues were marked with asterisks, and its hotspot proportion is about 0.412 (7/17) which is greater than the others 0.235 (8/34) (Figure 3A) and 0.320 (8/25) (Figure 3B). Thus, our results suggested that high rates of mutations were most likely to be found via integration process of covariance analysis and single-point mutations discovery compared with only using covariance analysis. Following the comparison, the identified residues with time-dependent covariances may play important roles during the course of HBV infection.

### Informative covariances correspond to clinical signatures

Plenty of studies from clinical or epidemiological researches on HBV investigation have claimed that specific amino acid mutations, especially in the regions of immunocyte epitopes, of HBV precore/core ORF encoding proteins would encompass the activities of cellular immune responses, such as B cell, T helper and cytotoxic T lymphocyte activations, for HBV DNA clearance in some liver-related diseases [12], [20], [30], [31]. However, these identified mutations were not derived from an analysis of massive time-varying sequences even if several publicly acceptable mutation hotspots had been reported. Hence, we had an insight into our identified time-dependent covariances and made some comparisons for four matched pairs of each case-control subgroup, such as the stationary pattern versus fluctuating pattern, declining pattern versus wavering pattern, HBeAg-seroconversion pattern versus HBeAg-nonseroconversion pattern and genotype B versus genotype C. Interestingly, in each pair comparison, the latter always have more complicated covariances stemming from more nodes and edges compared to the former. For example, the size (33) of fluctuating pattern time-dependent covariances is bigger than that of stationary pattern (3) (as shown in Figure 2). This result is certainly reliable because patients with stationary pattern have a stable serum HBV DNA level. Accordingly, HBV precore/core ORFs encoding proteins, HBeAg and HBcAg, were much easier to be recognized by the host's immune system [32]. In addition, the sizes of covariances are also correlated with the genotypes as well as HBeAg-seroconversion. It also has been reported that HBV infected patients in genotype C have a late HBeAg-seroconversion and more severe liver fibrosis compared to the genotype B [22], [33]. This consistent result indicated that the time-dependent covariances may not only contribute to serological outcomes but also play a functional role in the process of HBeAg-seroconversion [22]. Thus, our results corresponded to the previous studies and suggested that the complexity of covariances was strongly associated with viral kinetics, HBeAg-seroconversion and genotypes. To the best of our knowledge, these covariances for each subgroup have yet been reported. It may have some biological insights into a clinical enigma.

### HBV genotype C infections accompanied with a cyclic process of point mutation

In prior sections, our results have indicated that the various covariances were strongly associated with the patterns of viral kinetics, seroconversion and genotypes. This phenomenon was predominant in the time-dependent covariances, i.e. the residues have substitutions with different amino acid properties over time. Recently, some studies claimed that patients infected with HBV genotype B have a higher probability for HBeAg-seroconversion at an early stage compared to genotype C [22], [34], [35]. HBV genotype B could also cause a less progressive liver disease [34], [36]. Thus, different HBV genotypes would contribute to the outcomes of liver-related disease and virus persistence in the hosts. Here a question was raised whether the substitutions occur frequently in the patients with genotype B or C. Patients involved in the same subgroup were separated into two cohorts based on their HBV genotypes, B or C. We made the reasonable assumptions that HBV have a high activity in the early viral infection because at that time the viral foreign antigens have yet been recognized or even have yet been eliminated by host immune systems. Hence, for a given point mutation rule to one residue position, if a substitution (e.g., H→any kind of amino acids) took place at the baseline of the one year period and ultimately reversed to the original type of amino acid (e.g., any kind of amino acids→H) before the year-end time point period regardless of what kind of amino acids appeared during the intermediate steps, it was called “cyclic process”, whereas it was called “acyclic process”. According to this definition, the point mutations showed in column “Single-point mutations with different amino acid properties” in Table S2 that could be partitioned into several parts as shown in Table 3 and Table 4. It was obvious that the cyclic processes were predominant in the genotype C compared to B. Moreover, the genotype C also has a high proportion of mutation hotspots in cyclic processes (Table 3). For example, in the subgroup with wavering pattern, there were three hotspots out of seven single-point mutations (3/7) in cyclic process that was certainly higher than its corresponding control, genotype B (0/3). However, this phenomenon could not be observed in acyclic processes (Table 4). Taken together, our results suggested that biological alterations in precore/core amino acids of HBV genotype C have a higher complexity than that of genotype B. These results were consistent with previous studies. The identified single-point mutations with a cyclic process may also play important roles in HBV genotype C infection that have not been reported.

**Table 3:** Single-point mutations with different amino acid properties in cyclic process.

Subgroup types	Number of patients†	Genotype B	Number of patients††	Genotype C
Stationary pattern	3	34*, 104	3	
Fluctuating pattern	5	5, 44, 94	12	29*, 55, 56, 64, 89*, 109, 110, 116*, 159*, 164, 182*
Declining pattern	1		8	55, 56, 89*, 116*, 159*, 164
Wavering pattern	4	5, 44, 94	4	29*, 64, 109, 110, 159*, 164, 182*
HBeAg-seroconversion	3		3	56, 89*, 116*
HBeAg-nonseroconversion	2	5, 44, 94	9	29*, 55, 64, 109, 110, 116*, 159*, 164, 182*

HBeAg: Hepatitis B e antigen.

† : Number of patients in genotype B.

†† : Number of patients in genotype C.

*: Literature-reported mutation hotspots.

**Table 4:** Single-point mutations with different amino acid properties in acyclic processes.

Subgroup types	Number of patients†	Genotype B	Number of patients††	Genotype C
Stationary pattern	3		3	
Fluctuating pattern	5	34*, 67*, 89*, 108, 116*, 122, 164, 197	12	10, 34*, 42*, 56, 70, 92, 106, 116*, 126*, 142, 145, 147, 159*, 160, 192, 209
Declining pattern	1		8	10, 34*, 42*, 56, 70, 92, 106, 116*, 126*, 142, 145, 147, 159*, 192, 209
Wavering pattern	4	34*, 67*, 89*, 108, 116*, 122, 164, 197	4	56, 92, 159*, 160
HBeAg-seroconversion	3	34*, 67*, 89*, 197	3	10, 34*, 42*, 70, 116*, 126*, 159*, 209
HBeAg-nonseroconversion	2	34*, 108, 116*, 122, 164	9	56, 92, 106, 116*, 126*, 142, 145, 147, 159*, 160, 162

HBeAg: Hepatitis B e antigen.

† : Number of patients in genotype B.

†† : Number of patients in genotype C.

* : Literature-reported mutation hotspots.

### Development of a tree-based visualization for an early prediction

According to the virological and serological patterns stated in the section of patients and study design, a HBV infected patient can be categorized into stationary or fluctuating patterns followed by declininig, wavering, HbeAg-seroconversion or HbeAg-nonseroconversion patterns. However, patients undergo early pharmaceutical interventions which can mitigate unwanted clinical symptoms and prolong the duration of chronic active liver disease. Thus, a potentially powerful prediction at early HBV infection is required.

**Figure 4:**
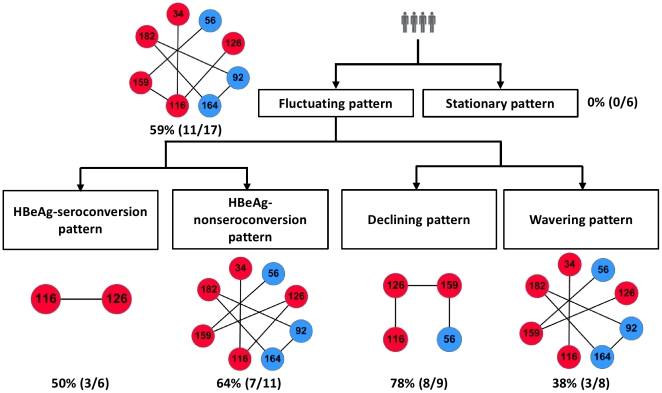
A tree-based visualization flow. Each rectangular box represents the subgroups. Representative covariances are next to each box. Numerator and denominator showed in parentheses indicate covered and intra-subgroup patient numbers respectively.

In this section, we introduced another approach on an attempt to predict the serological or virological outcomes at an early stage of HBV infection. Covariance analysis was applied on the sequences derived from the early stage spanning from baseline, t_1_, to maximal serum HBV DNA time point preceding the nadir, t_p_, (middle right in Figure 1). The identified early-stage covariances were subsequently used to determine the intersections with aforementioned time-dependent covariances (bottom left in Figure 1). All the processes followed the principle of our proposed leave-one-out-like cross-validation – see also Methods. For increasing the feasibility of our results to clinical application, displaying data in a readily comprehensible fashion to clinicians is required. The representative covariances of each subgroup in this study were utilized to develop a tree-based visualization flow in Figure 4. Most subgroups have more than half coverage rates. This flow may provide a useful information to clinicians and it is also a simple application on the clinical diagnosis to predicting what kind of patterns for patients who will be exhibited at the terminal stage. For example, when a new HBV-infected patient comes to a hospital, a covariance analysis will be applied on their amino acid sequences. Then the resultant covariances will be checked whether they are covered by any possible covariances from top to bottom of the tree. Clinicians can prescibe the patient a suitable treatment for avoiding the development of an aggressive liver disease. On the other hand, it is worth noting that both HBeAg-seroconversion and HbeAg-nonseroconversion subgroups had an identical covariance of residue 116 and 126. To have further insights into the characteristics of covarying pairs of amino acids, the pairs were counted from both subgroups. The pair G-L and S-I were predominant in HBeAg-seroconversion and HbeAg-nonseroconversion respectively. A significant distribution was examined by using Chi-square test (p<0.0001, data not shown). These two sites not only had been identifed as hotspots but also could be considered as a good biomarker of the two subgroups in early HBV infeciton. Therefore, in this section, the interesting results not only provided a potential clinical diagnosis prediction but also showed several novel covariances of most subgroups.

## Discussion

In this study, we utilized an integration of covariance analysis and a mining single-point mutation method on a massive monthly serum-derived HBV precore/core amino acid sequences to identify several representative covariances of each subgroup with various serological or virological patterns. This prospective study has many advantages since we integrated statistical analyses, computing techniques and large-scale viral sequences over a yearlong observation period. This was different from previously traditional approaches; clinical numeric data retrieved from patient samples were easier to be analyzed by clinicians or statisticians. However, to date, there are still many problems are left unresolved due to high mutation rates of viruses. A lot of information hidden in the viral sequences is also important to the interplay between viruses and infected patients. For example, which mutation residue of HBV precore/core amino acids is mainly responsible for escaping attack from the host immune system even if a small number of mutation hotspots have been identified? This is the main reason we attempted to have insights into the amino acid alterations affecting the outcomes of HBV-infected patients in this work.

Since the replication of HBV DNA in human hosts is error-prone [20], from a clinical point of view, clinicians cannot easily predict or find out the exact amino acid mutations or underlying mechanisms to the high diversity of clinical syndromes. Predicting and/or discovering the specific single or multiple mutations associated with the outcomes of HBV infection is a challenge [32]. An analytical approach to amino acid sequences is indispensable to answer the question. In addition, it is well known that two serum-derived proteins, HBeAg and HBcAg, not only can be encoded by precore/core ORF but also have been regarded as two main indicators of active HBV replication. The expressions of the foreign antigens could be altered through a DNA transcriptional regulation with cis- or trans-mechanisms. In the previous cases, plenty of studies have reported that the consequences of mutations in basal core promoter were associated with the liver disease and risk of HCC development even after adjusting for HBV genotypes [37], [38]. However, it cannot illustrate how protein quality changes influence HBV persistence; instead, it only focused on the protein quantity alterations. Only a small proportion of studies focused on the amino acid mutations of HBV precore/core or other ORF [32], [39], [40]. It has been reported that 13 significant mutation hotspots were located in MHC class II-restricted T cell epitopes in host in hepatomas [16]. These hotspots were considered as a ground truth for evaluating the identified covarying residues. Figure 3C showed a higher hotspot precision compared to only applying covariance analysis method as shown in Figure 3A. This result indicated that our integration method may have more chances to provide more biological insights into genetic regulations of HBV. The covarying residues presented in Figure 3C were covered by three well-known mutation clusters located on precore/core region including regions 77–89, 113–130 and 176–184 [41]–[43]. Specifically, an Asian study claimed that three most frequent found hotspots including L89V, S116G and I126L were responsible for HBV escaping immune response in Chinese with chronic hepatitis B [44]. These hotspots also could be identified by our integration method. In order to further verify if our results are credible, the literature reports on three class II HLA restricted T cell epitopes, spanning amino acids 29–49, 79–98 and 146–160, within precore/core protein identified by Penna et al. [45] and our findings including mutation position 34, 44, 89, 94 and 159 were congruent. Moreover, we also discovered several multiple mutations 55, 56, 159 and 164, that were located on class I HLA restricted T cell epitopes, spanning amino acids 47–56, 159–169 and 170–180, [46], [47]. It is worth noting that three amino acid positions 34, 55 and 94 within human HLA restricted T cell epitopes have yet been reported. These mutations could be regarded as substantial hotspots to the course of HBV infection. Besides, there were also some putative mutations exhibited in our results such as position 64, 70, 104 and 108, even if it have not been regarded as main targets of immunocyte recognition. Taken together, the mutations discovered by our proposed integration method corresponded highly with antigen expression in the context of cellular immune-recognized epitopes. These findings may lead to a dramatically conformational change of HBeAg and/or HBcAg structures during the period since covariance analysis had been used to identify the residue pairs in protein 3-dimentional structures [25], [48].

Another important issue in epidemiology is the viral activities in patient. Infections were often of high persistence due to highly dynamic mutations in viral genomics or different genotypes [49]. In the latter case, HBV genotype B induced a greater T helper cell 1 response than T helper cell 2, compared to genotype C during hepatitis flares in HBeAg-positive patients [35]. However, to date, the underlying mechanisms have yet been elucidated completely. In this prospective study, all samples and serum-derived HBV precore/core ORFs were directly sequenced and translated into amino acids from HBeAg-positive chronic B carriers. In our previous work, we had presented a quick and useful method using two-step melting curve analysis for genotyping of HBV genomic sequences [29]. Different genotypes may associate with the certain mutations through mining single-point mutation rules from time-varying sequences; genotype C had an overwhelming majority of cyclic processes. This interesting result hinted that the original amino acids of HBV genotype C may have a higher probability of reappearance. In other words, HBV genotype C has a higher adaptability compared to the genotype B. Checking with the previous literature, a large number of researches showed that the genotypes of HBV were strongly associated with the chance of HBeAg-seroconversion, recovery rates of liver-related diseases, and patterns of viral kinetics [22], [33], [35], [50], [51]. For example, HBV genotype C takes a more aggressive disease course than genotype B in HBeAg-positive patients [50]. Our results corresponded highly to the previous studies. In Table 3, the genotype C has a larger number of cyclic mutations and literature-reported mutation hotspots compared to B during this one year period, we reasonably inferred that HBV genotype C has a higher ability to escape the host immune system responses. Moreover, these changes were accompanied with changes in amino acid properties, which may lead a dramatically conformational change for the protein structure(s). It is worth noting that the identified single-point mutations have yet been reported, which may be novel immunocyte recognition targets for developing targeted therapies in the future.

This study has several advantages. We improved the drawback of only using covariance analysis method via integrating additional information, such as single-point mutation rules with different amino acid properties from patient monthly serum-derived sequences. Moreover, we developed a tree-based visualization flow chart which could be regarded as a useful tool to predict the serological or virological outcomes in an early HBV infection stage (Figure 4). Interestingly, the coverage rate of subgroup with stationary pattern is zero, whereas most remaining covariances have more than half coverage rates. To the best of our knowledge, stationary pattern have a serologically stable HBV DNA level due to no specific mutation sites. Therefore, the tree-based visualization flow chart is applicable to clinical diagnosis and is valuable for clinicians to prescribe patients appropriate treatment before symptomatic evidence appears. Our proposed method could be widely applied on sequences of other species even if the sequence length is not long.

## Supporting Information

Figure S1 **An example of mining single-point mutations.** CS: Conserved sequence.
(TIF)

Figure S2 **An example of leave-one-out-like cross-validation.**
(TIF)

Table S1 **Statistical examinations of all covariances.**
(XLS)

Table S2 **Single-point mutations of all subgroups.**
(XLS)

Table S3 **Single-point mutation rules of all patients.**
(XLS)

Text S1 **A list of 13 literature-reported mutation hotspots.**
(TXT)
