# Supplementary figures and images for: Analysis of Precore/Core Covariances Associated with Viral Kinetics and Genotypes in Hepatitis B e Antigen-Positive Chronic Hepatitis B Patients
Source: PLoS One. 2012 Feb 27;7(2):e32553. doi: 10.1371/journal.pone.0032553 (PMC3288105; doi:10.1371/journal.pone.0032553)

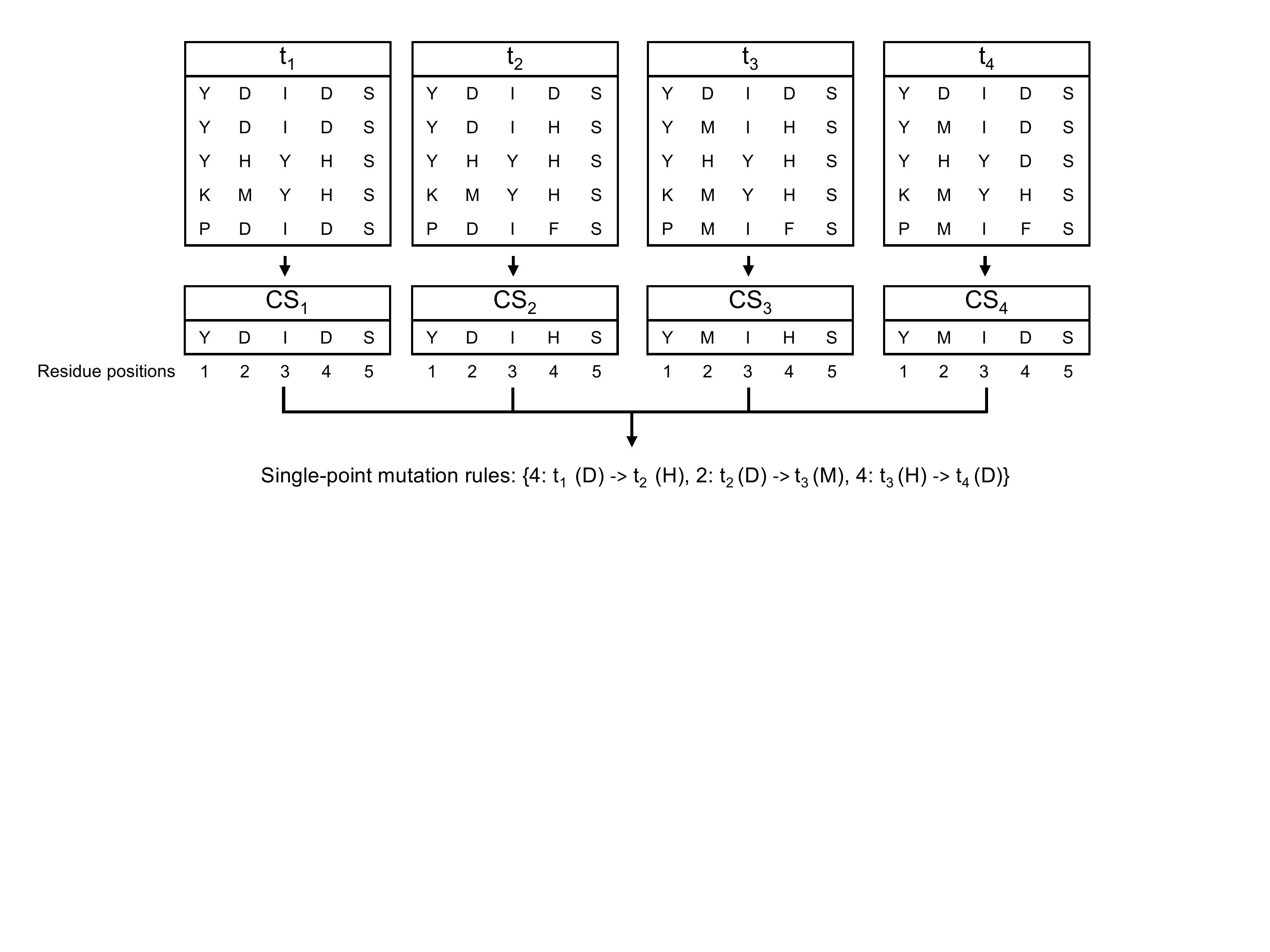

Supplement: Figure S1 — An example of mining single-point mutations. CS: Conserved sequence. (TIF) [file pone.0032553.s001.tif]

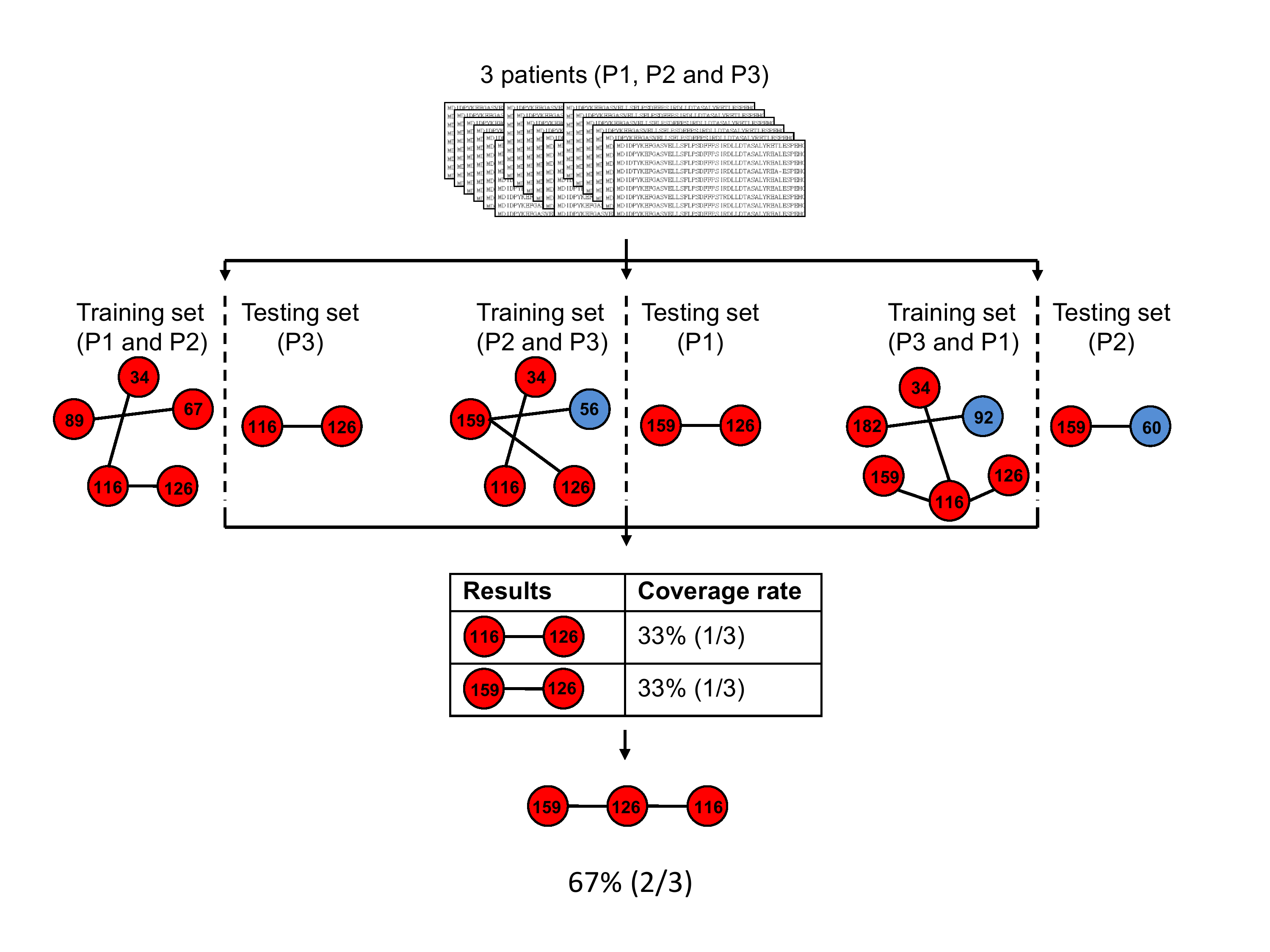

Supplement: Figure S2 — An example of leave-one-out-like cross-validation. (TIF) [file pone.0032553.s002.tif]
